# Supplementary figures and images for: Regulated targeting of the monotopic hairpin membrane protein Erg1 requires the GET pathway
Source: J Cell Biol. 2022 May 19;221(6):e202201036. doi: 10.1083/jcb.202201036 (PMC9123286; doi:10.1083/jcb.202201036)

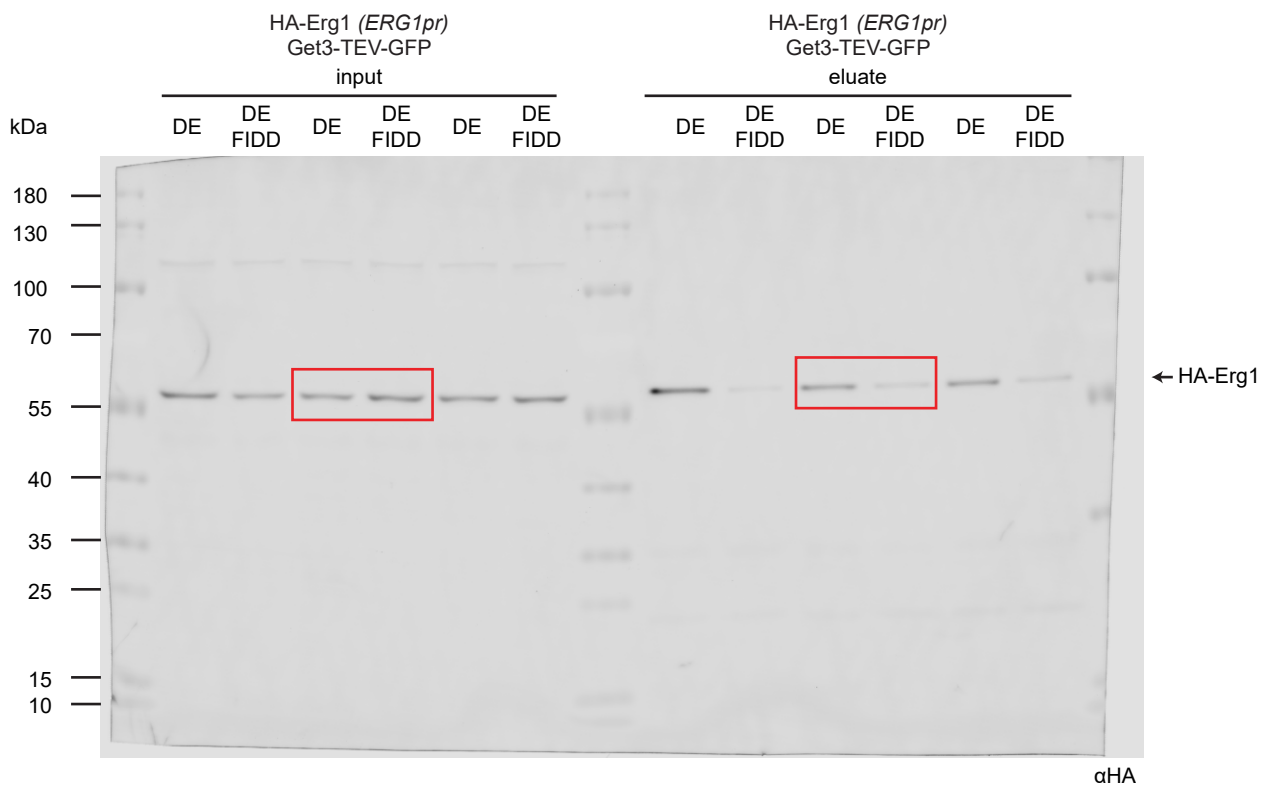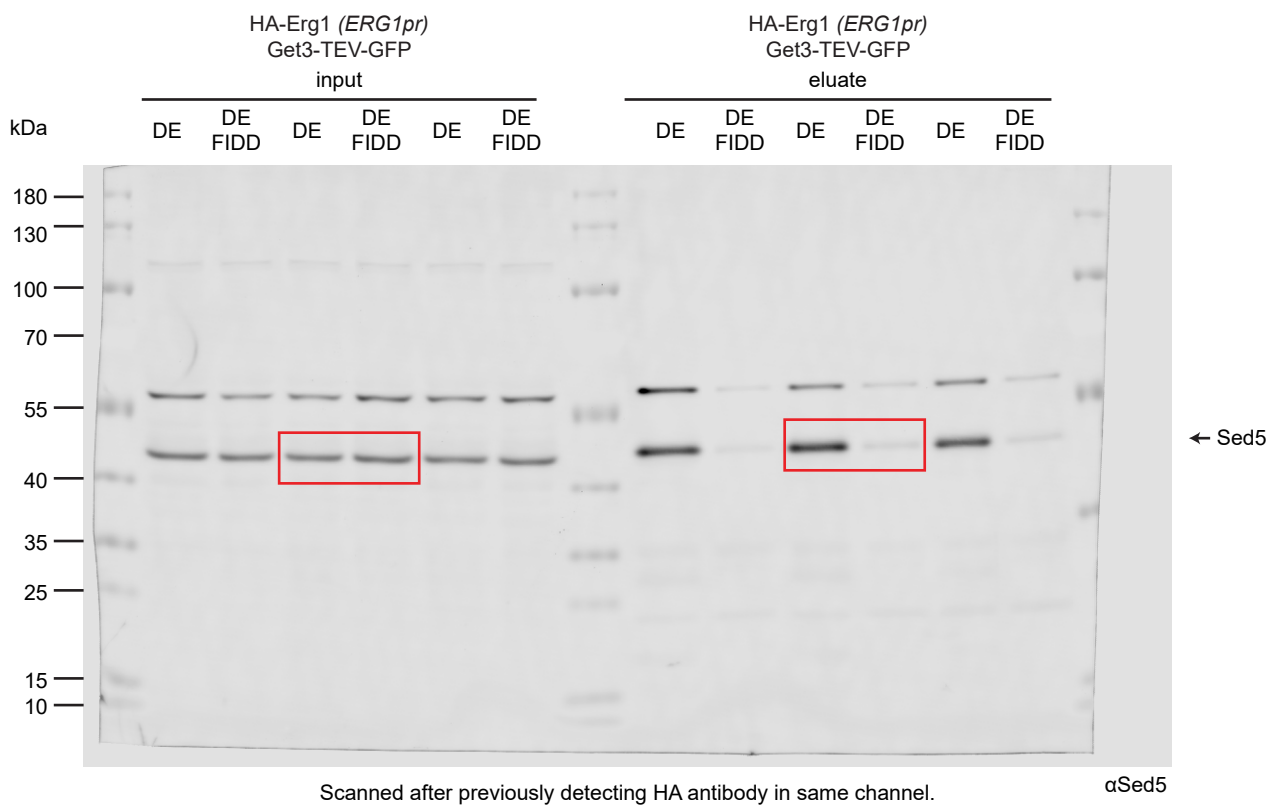

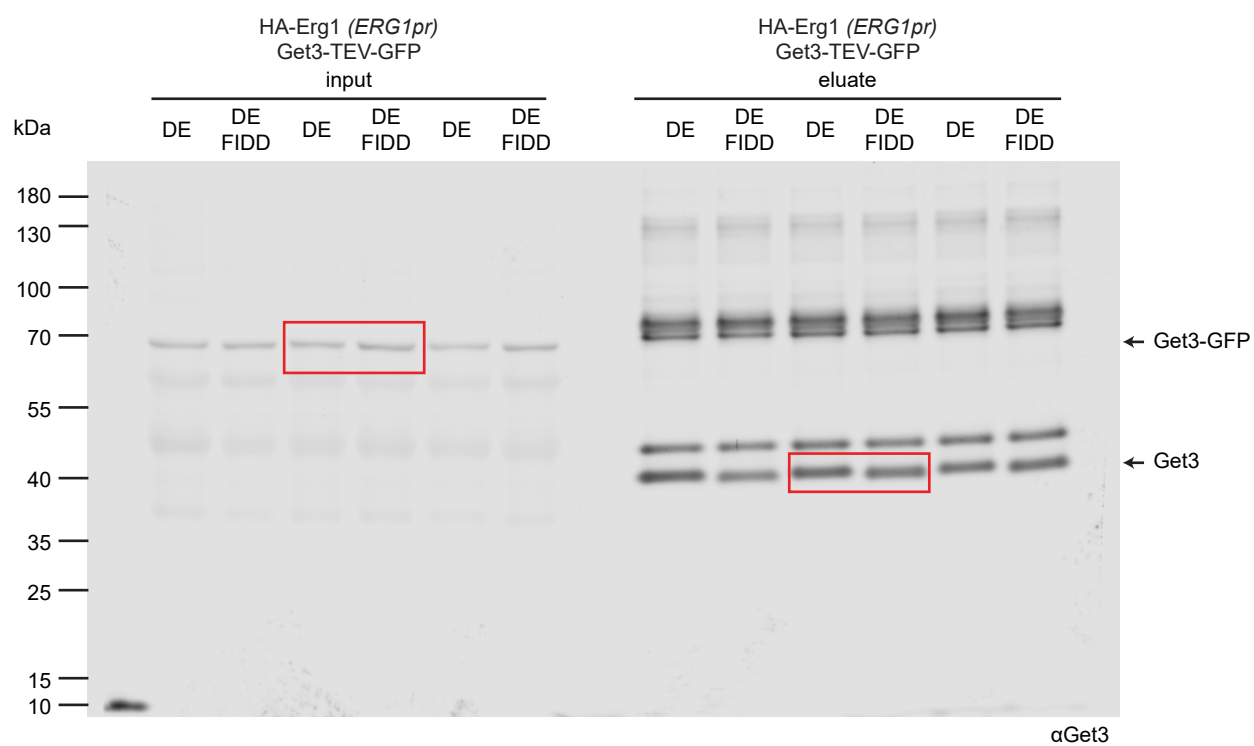

Supplement: SourceData F2 — is the source file for Fig. 2. [file JCB_202201036_SourceDataF2.pdf]

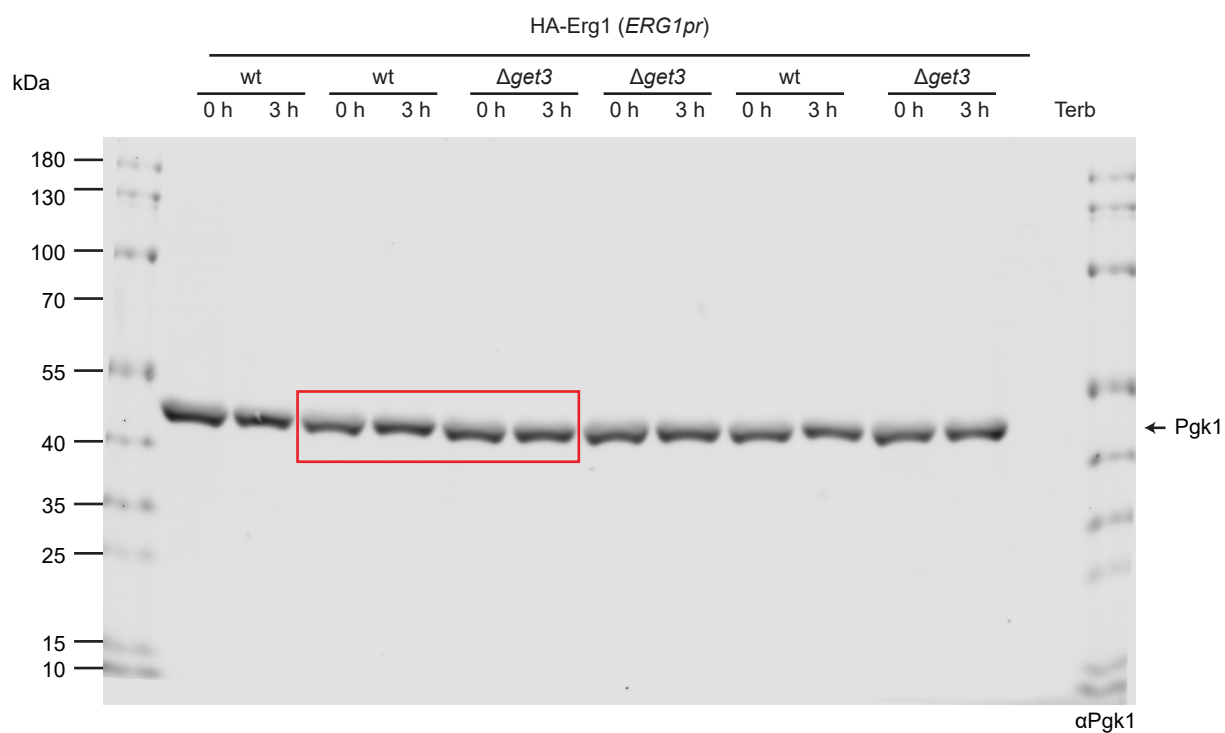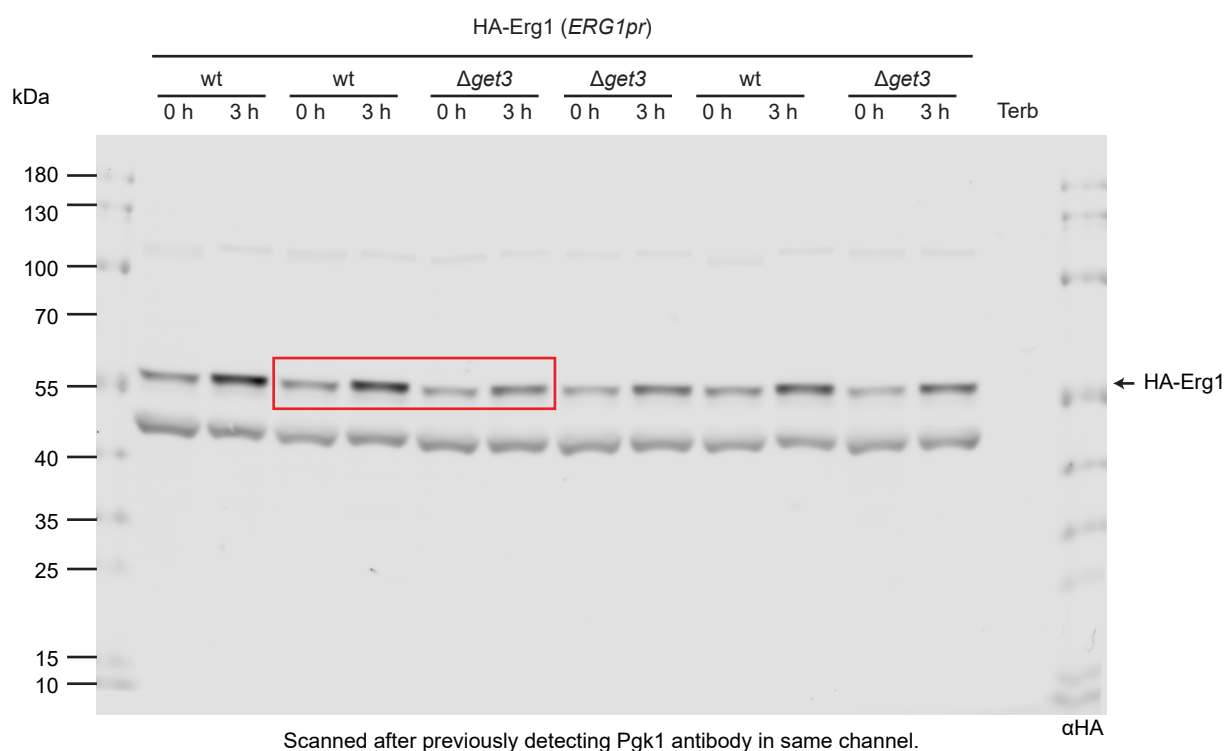

Supplement: SourceData F4 — is the source file for Fig. 4. [file JCB_202201036_SourceDataF4.pdf]

Figure 5C

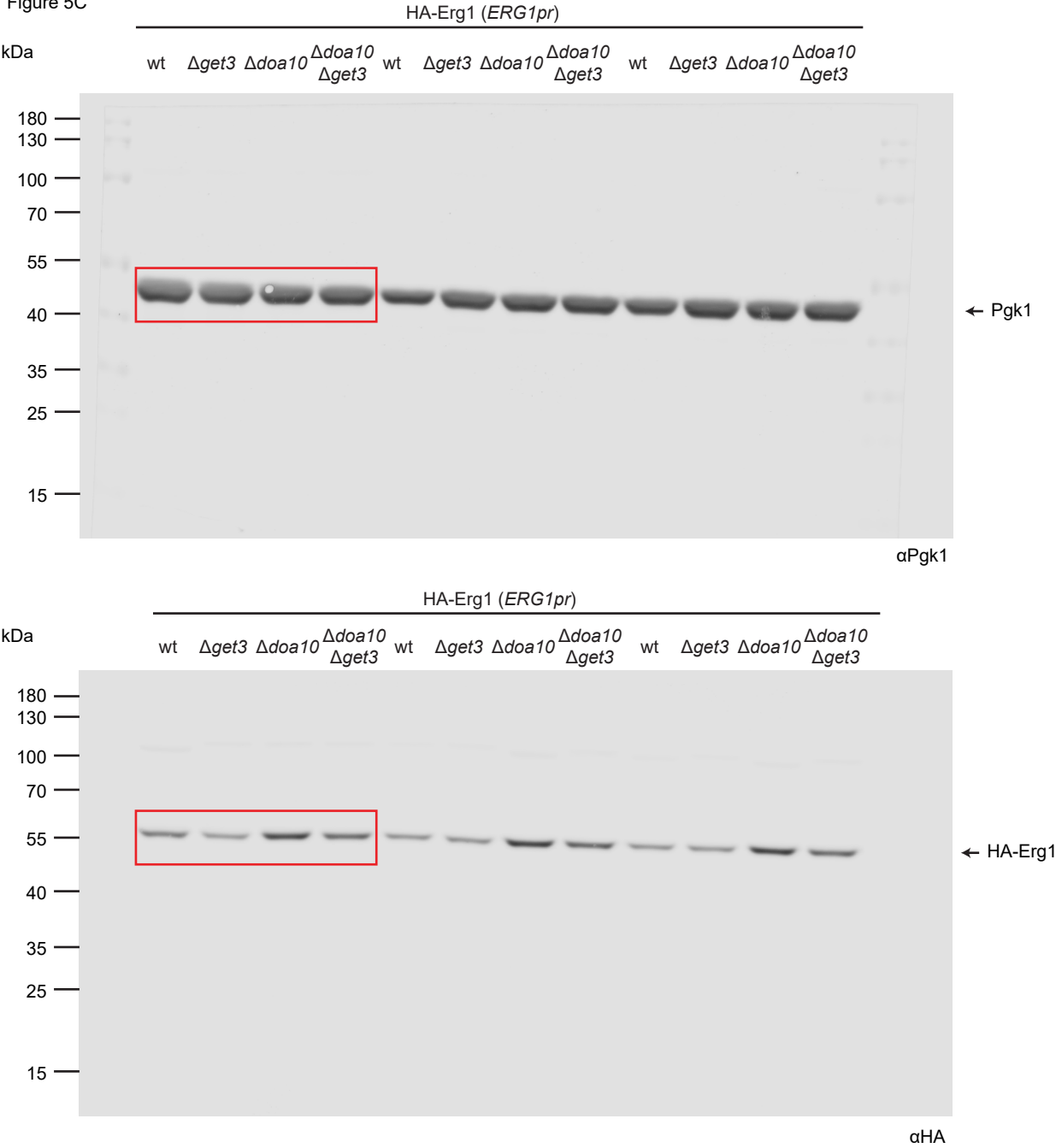

Figure 5E

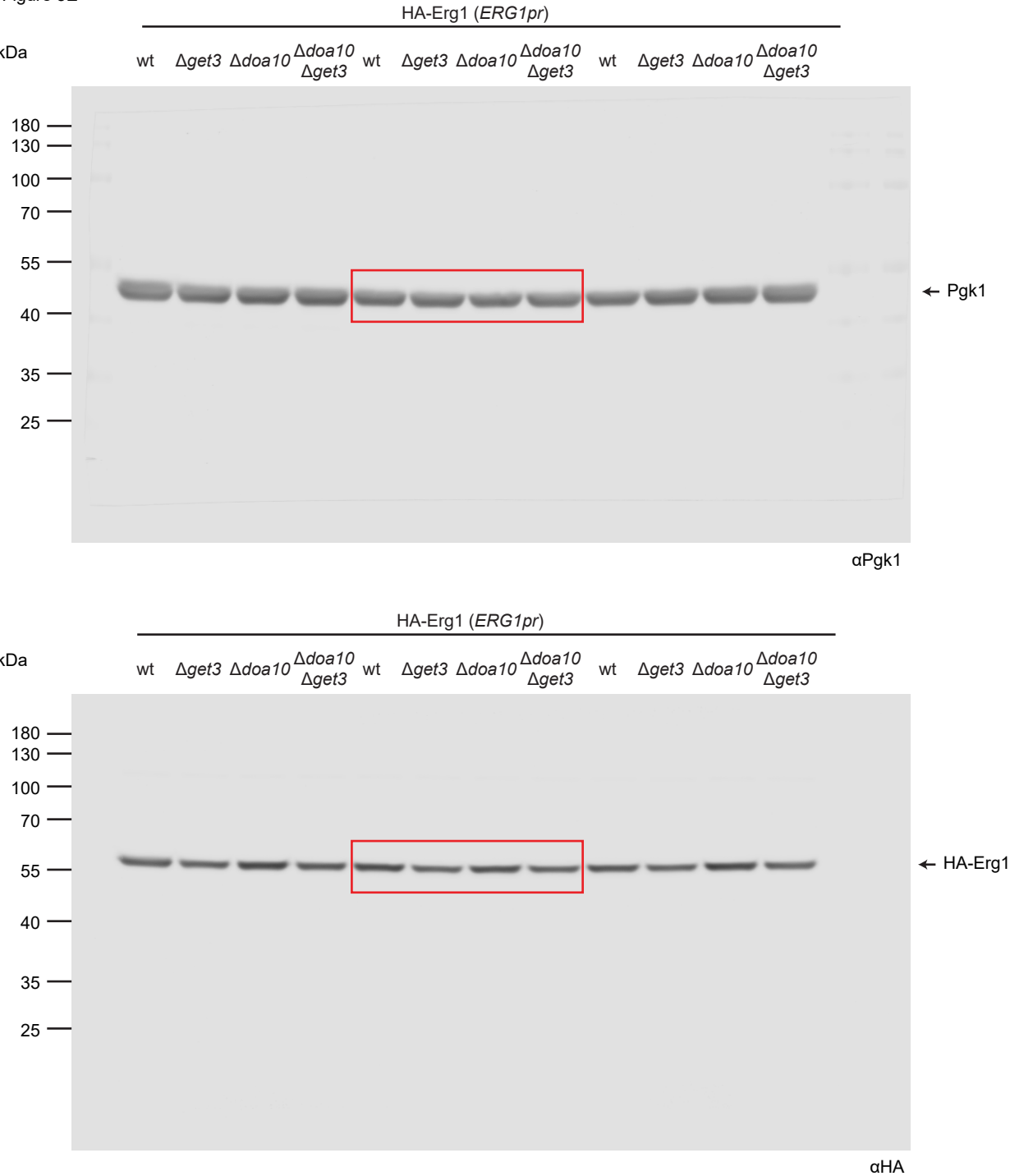

Supplement: SourceData F5 — is the source file for Fig. 5. [file JCB_202201036_SourceDataF5.pdf]

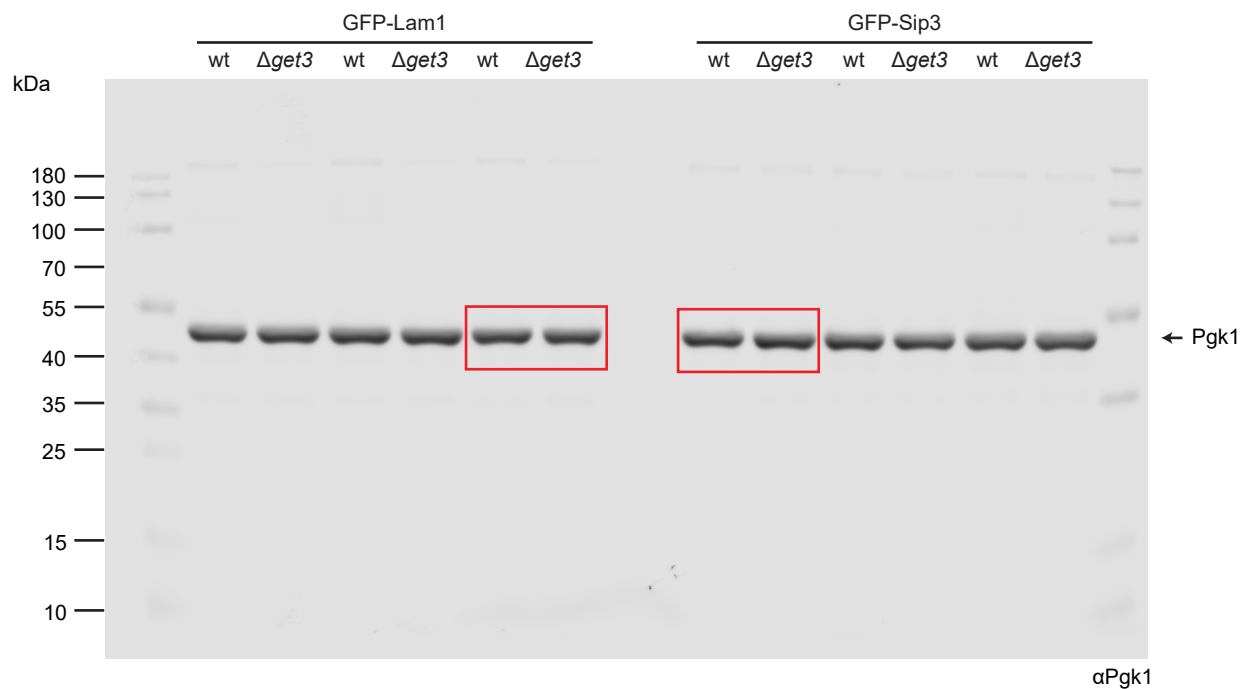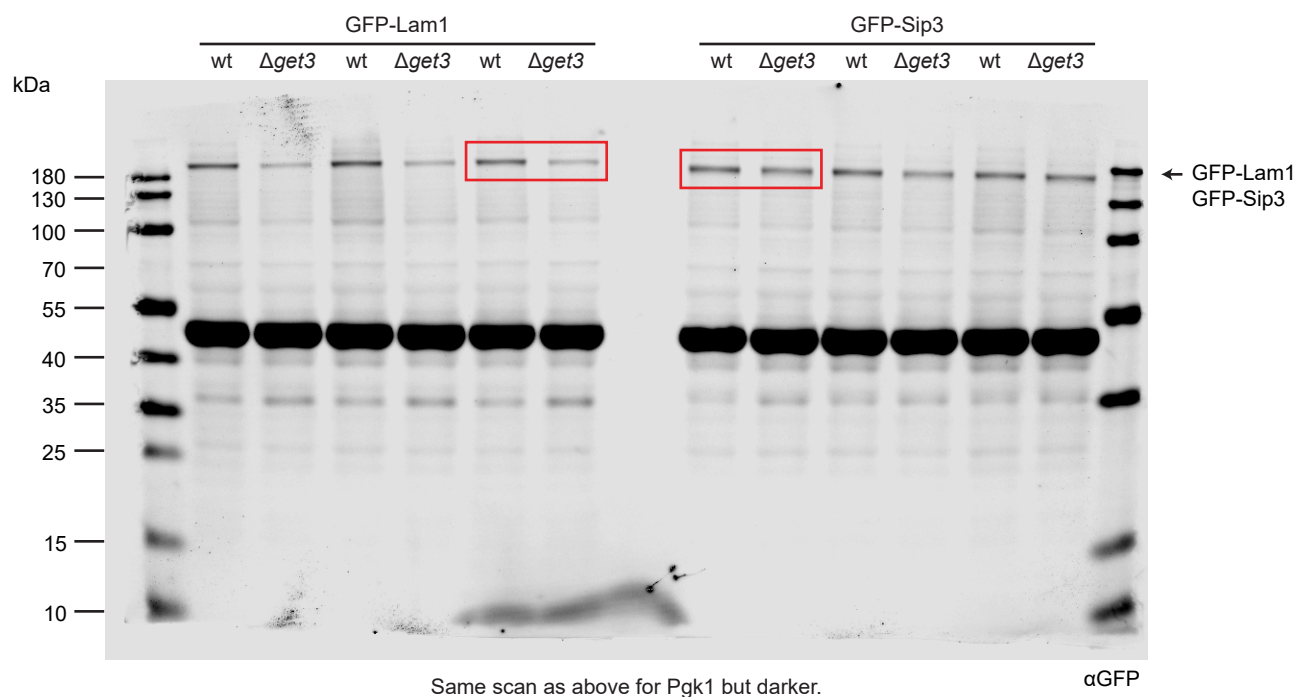

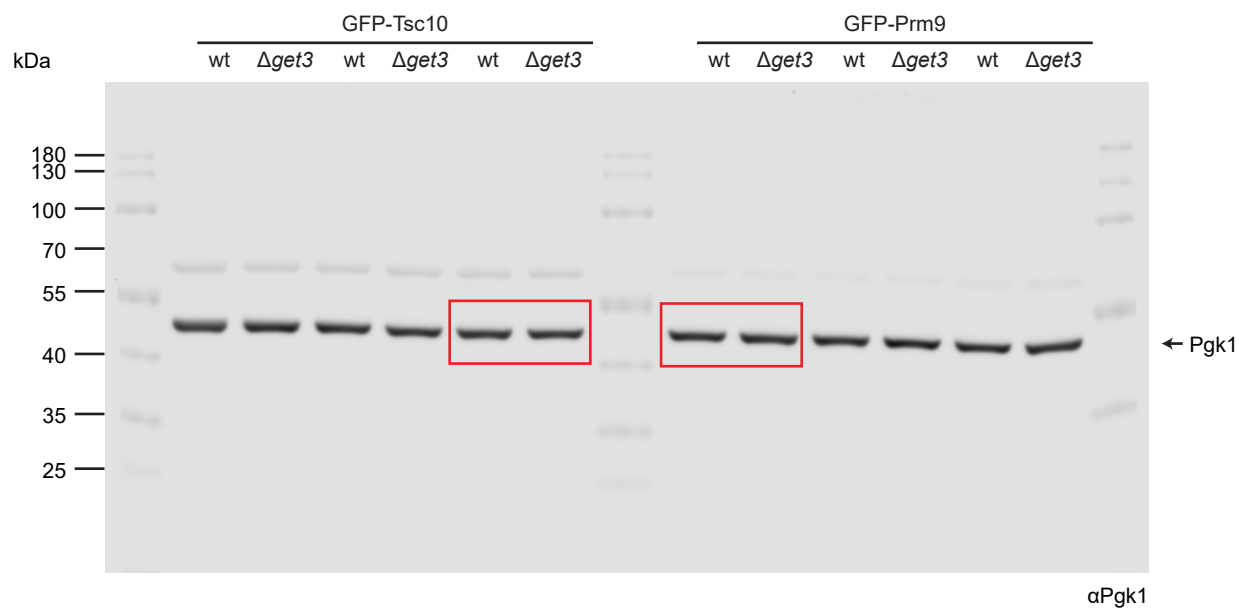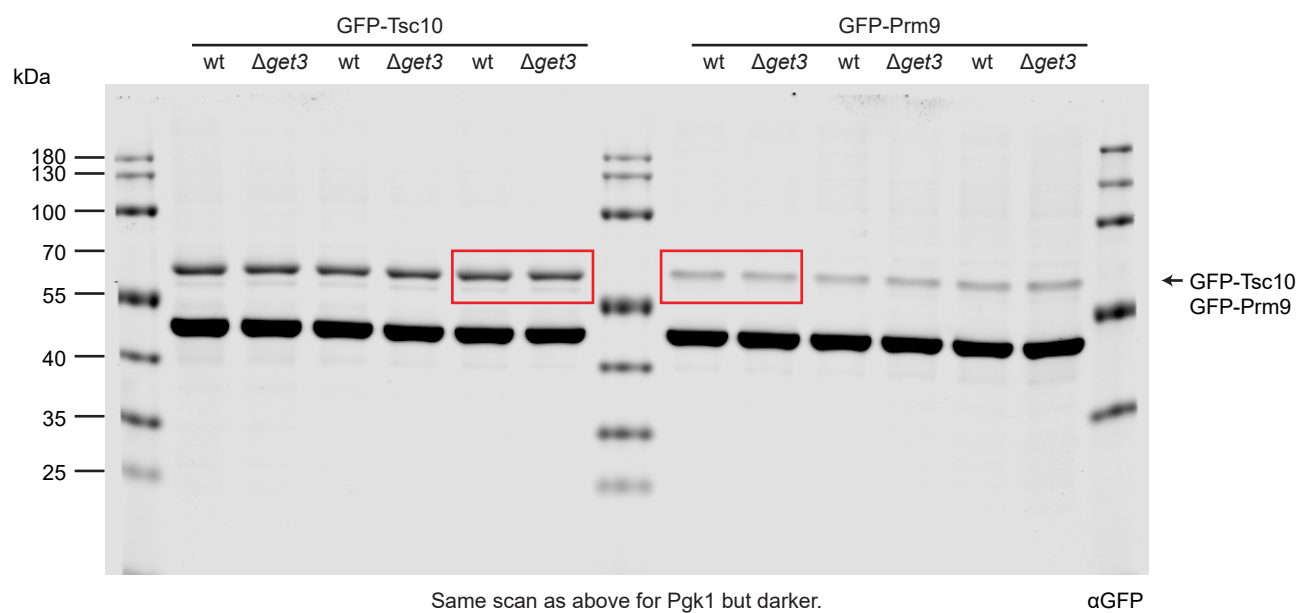

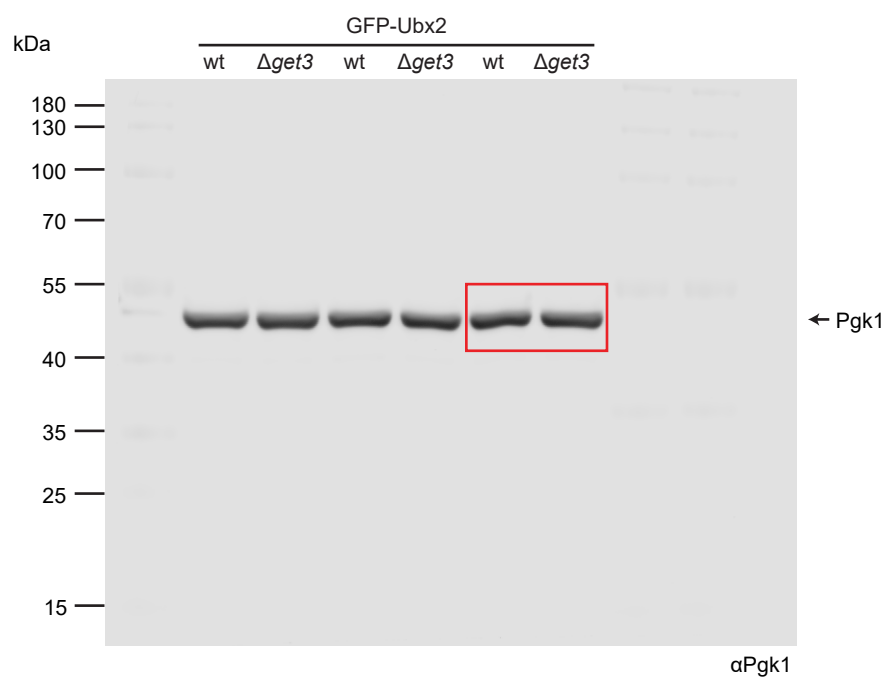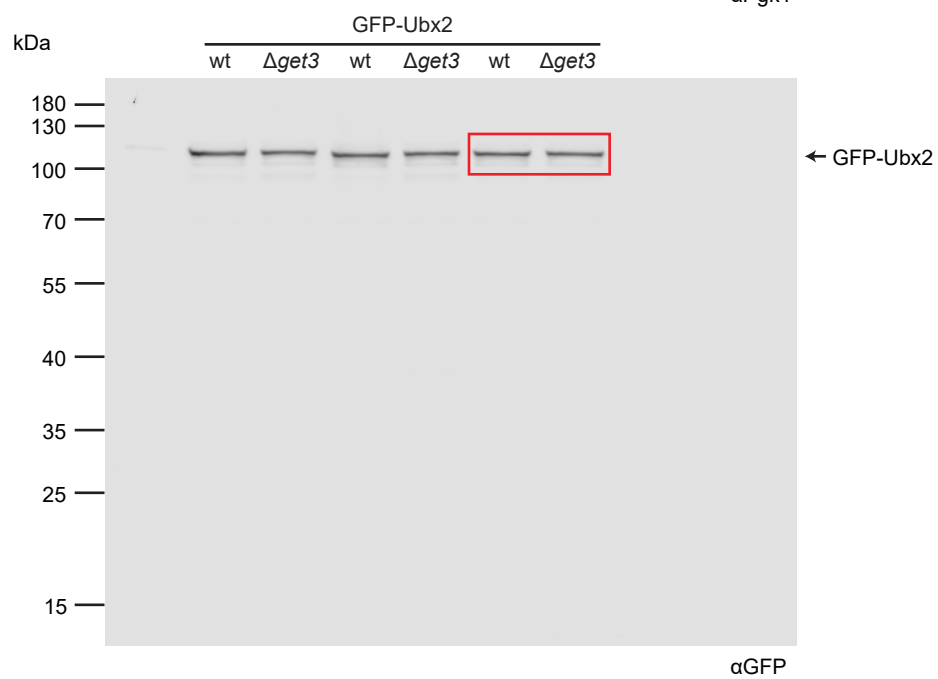

Supplement: SourceData F7 — is the source file for Fig. 7. [file JCB_202201036_SourceDataF7.pdf]

Figure S4C

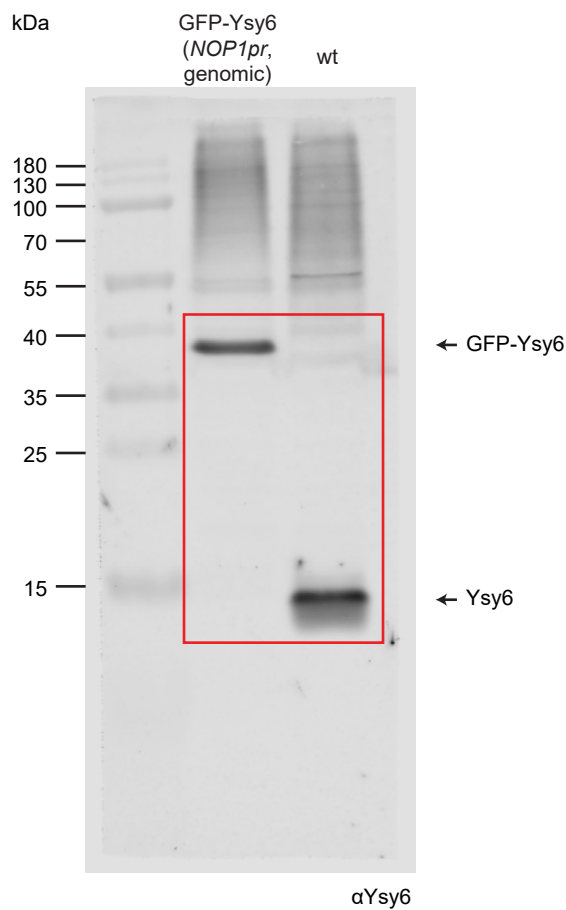

Supplement: SourceData FS4 — is the source file for Fig. S4. [file JCB_202201036_SourceDataFS4.pdf]
