# Supplementary material for: Regulated targeting of the monotopic hairpin membrane protein Erg1 requires the GET pathway
Source: J Cell Biol. 2022 May 19;221(6):e202201036. doi: 10.1083/jcb.202201036 (PMC9123286; doi:10.1083/jcb.202201036)
Supplement: SourceData F3 — is the source file for Fig. 3. [file JCB_202201036_SourceDataF3.pdf]

Figure 3D

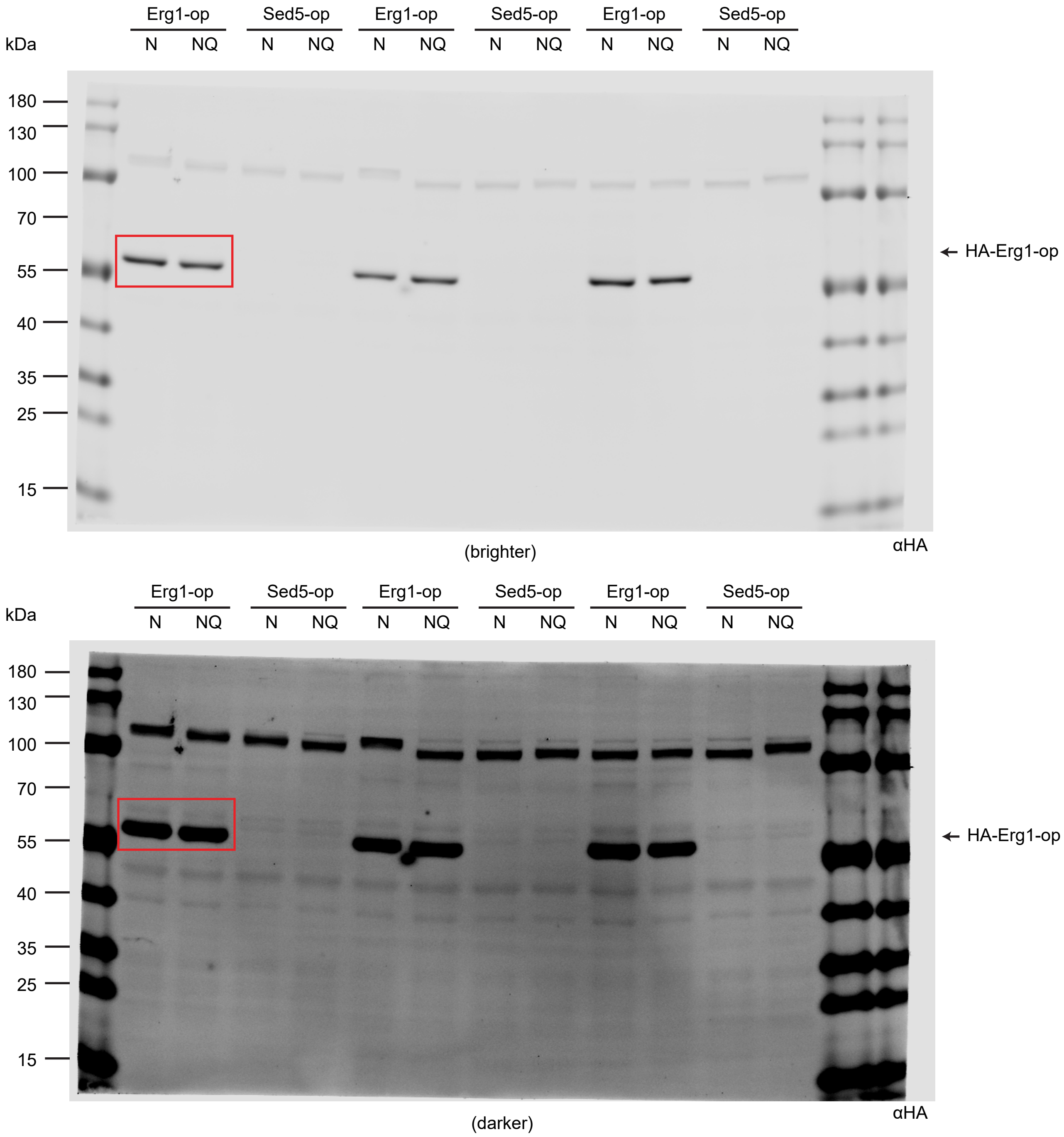

Erg1-op
Sed5-op
Erg1-op
Sed5-op
Erg1-op
Sed5-op
kDa
N NQ N NQ N NQ N NQ N NQ N NQ
180
130
100
70
55
40
35
25
15
← HA-Erg1-op
(brighter)
αHA
Erg1-op
Sed5-op
Erg1-op
Sed5-op
Erg1-op
Sed5-op
kDa
N NQ N NQ N NQ N NQ N NQ N NQ
180
130
100
70
55
40
35
25
15
← HA-Erg1-op
(darker)
αHA

Figure 3D

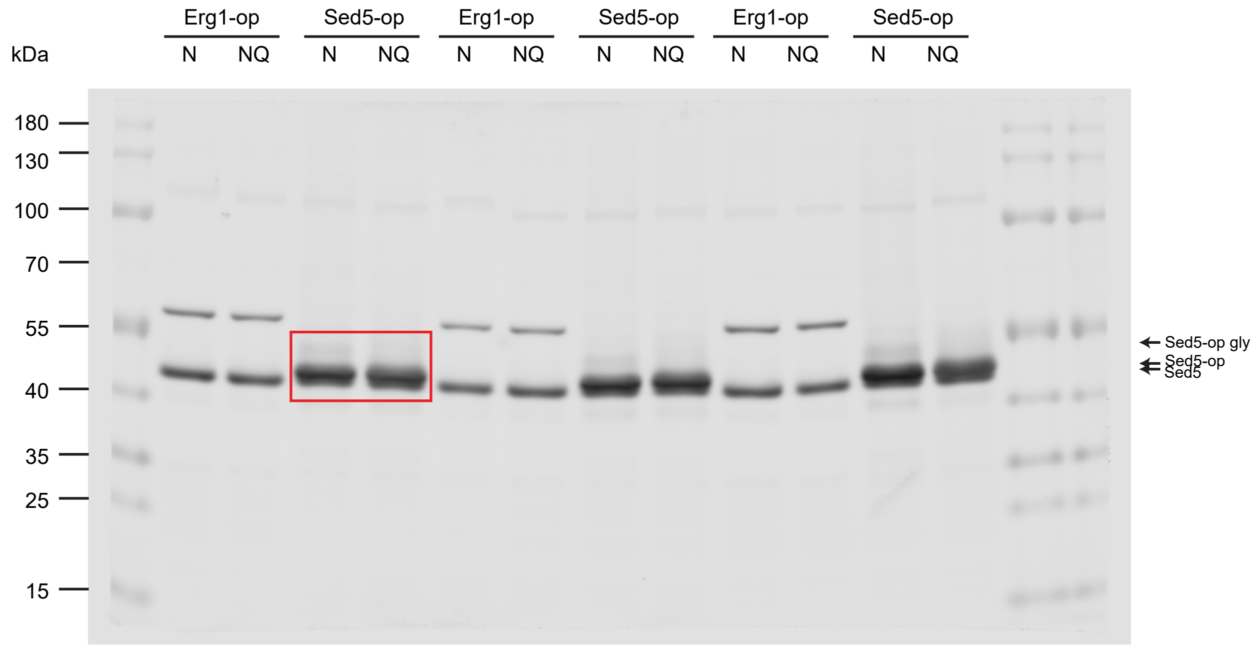

(brighter, scanned after previously detecting HA antibody in same channel)

αSed5

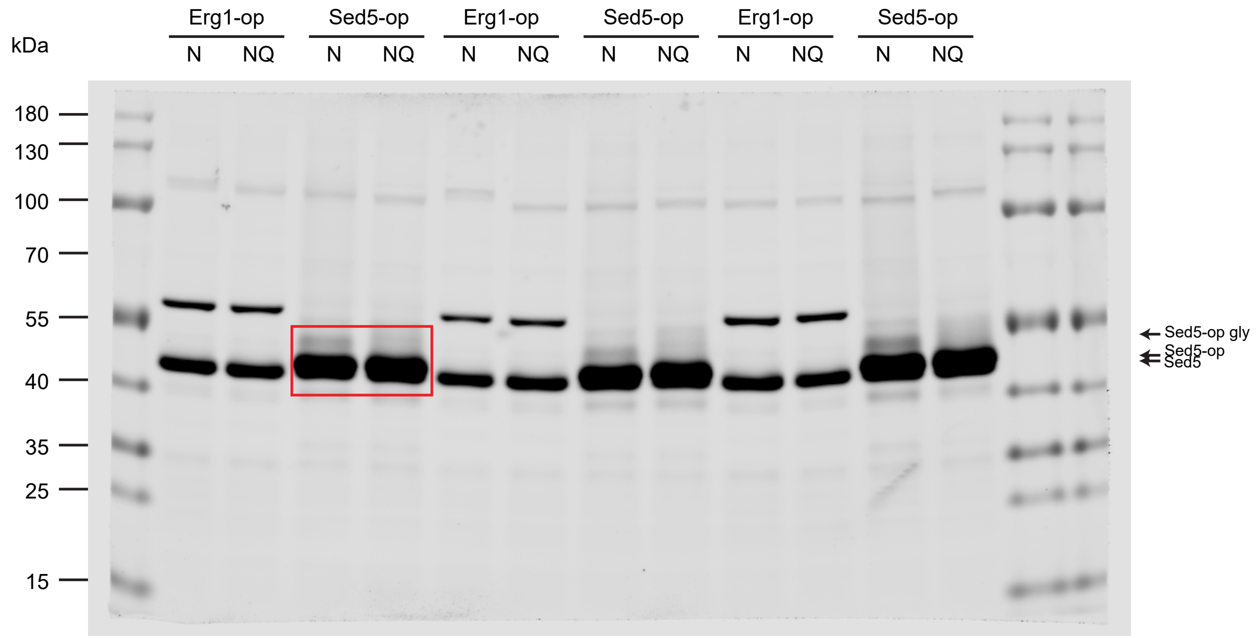

(darker, scanned after previously detecting HA antibody in same channel)

αSed5

Figure 3D

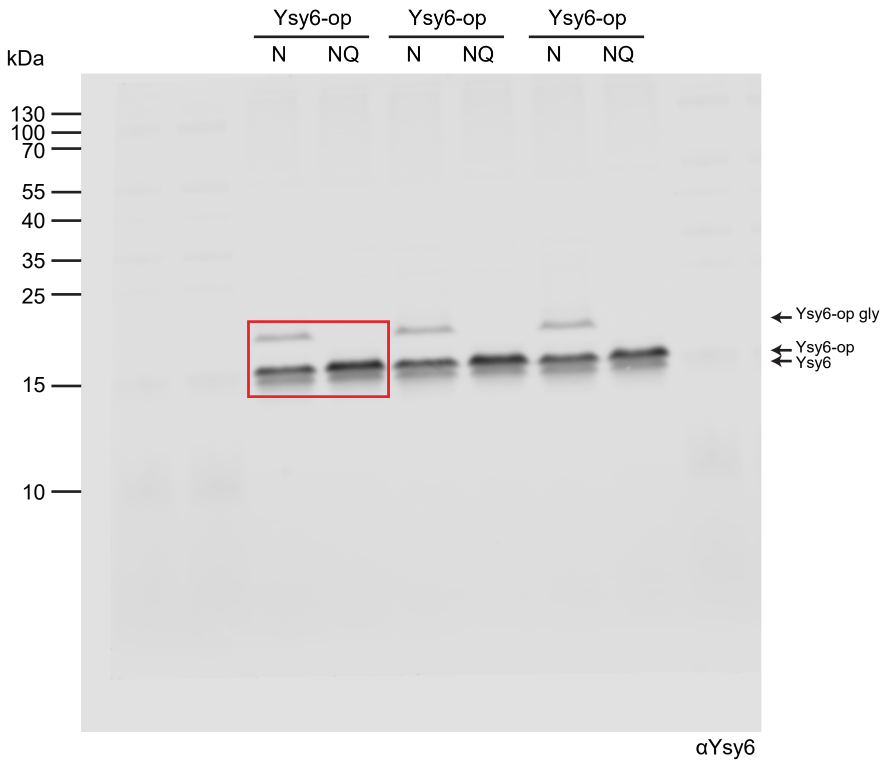

Figure 3E

Replicate 1

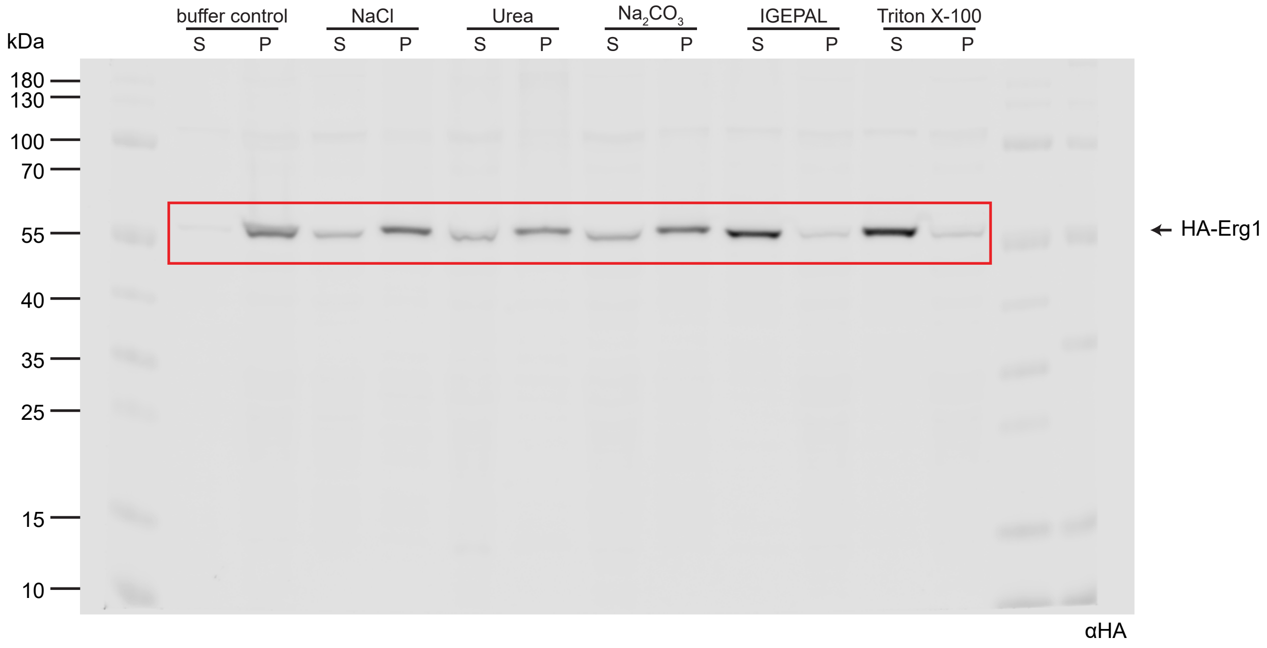

Figure 3E

Replicate 1

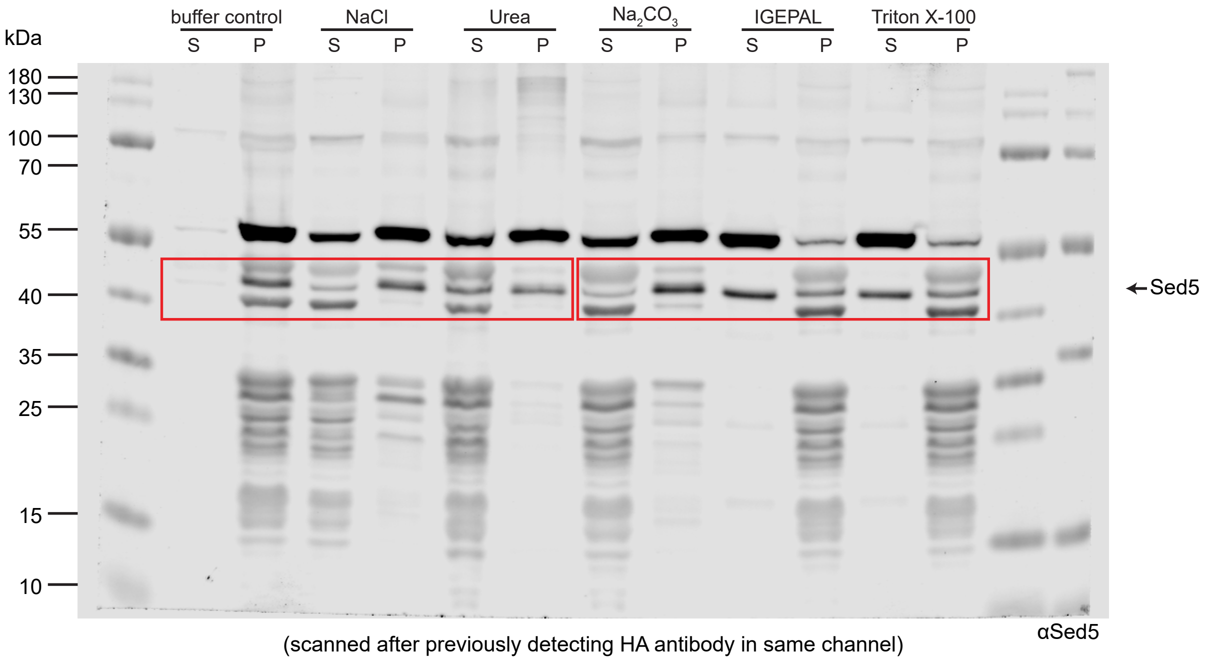

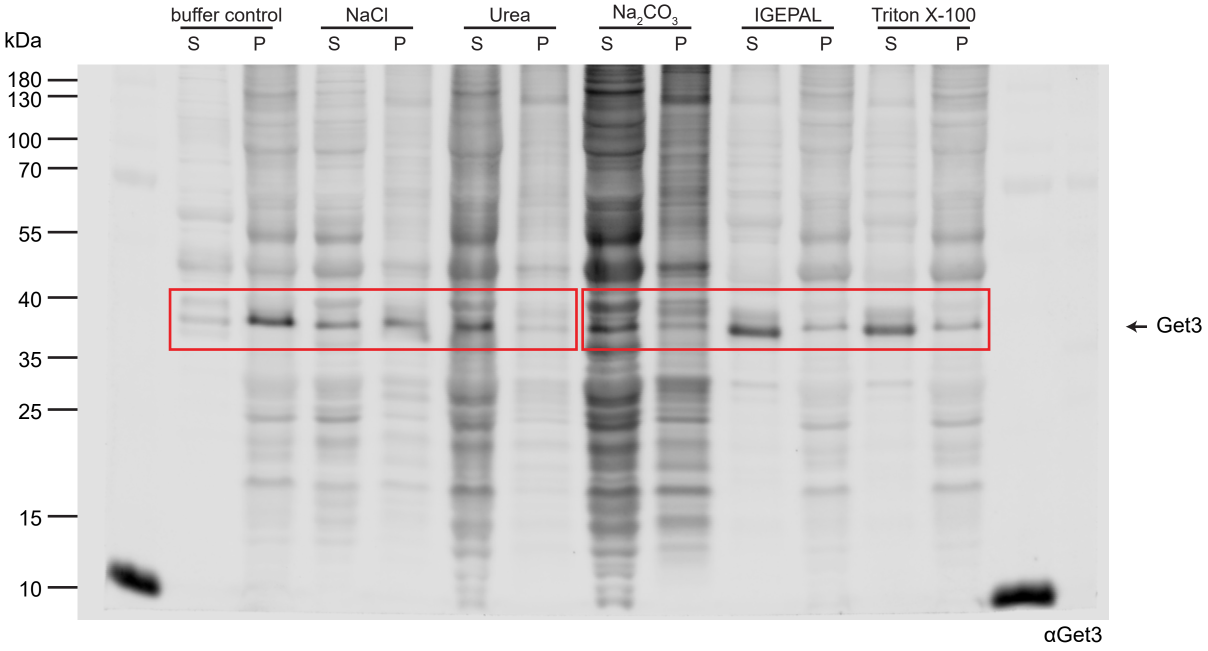

Figure 3E

Replicate 2

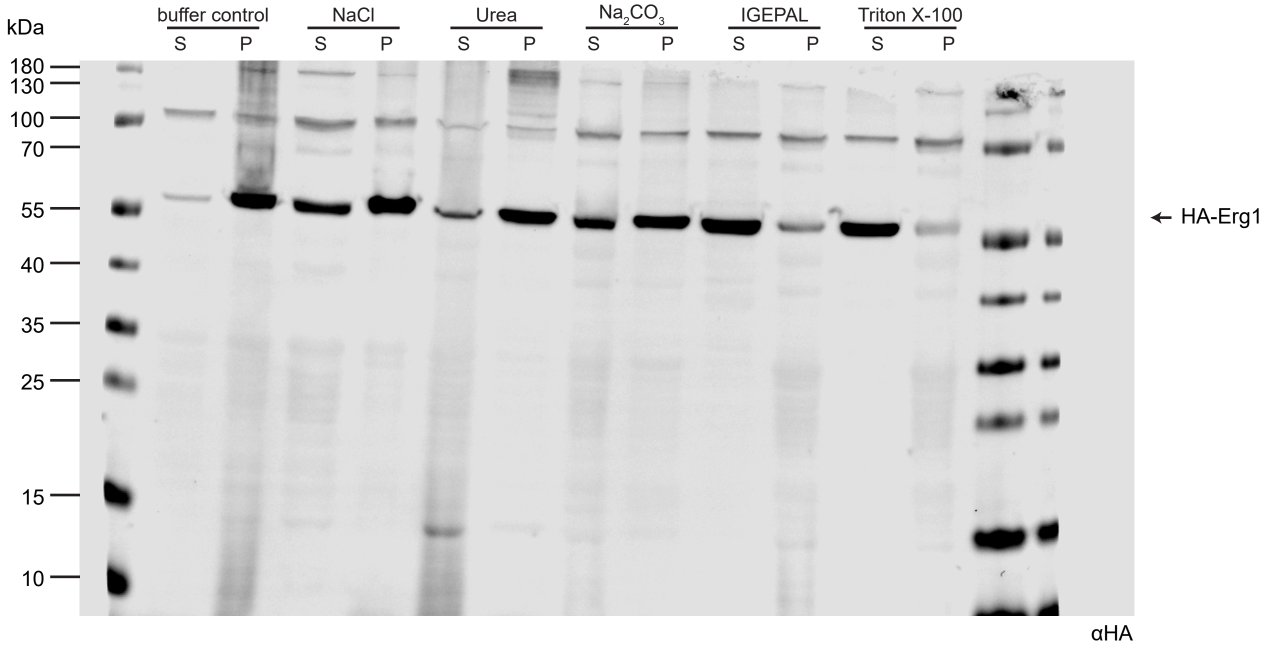

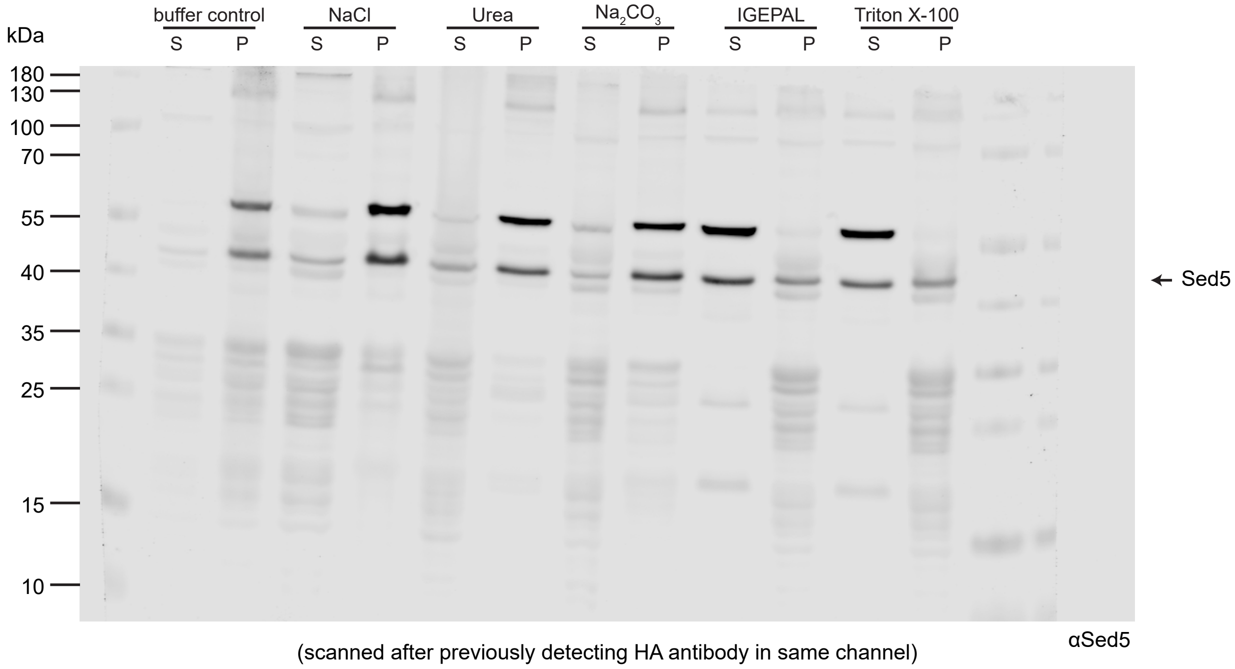

(scanned after previously detecting HA antibody in same channel)

Figure 3E
Replicate 2

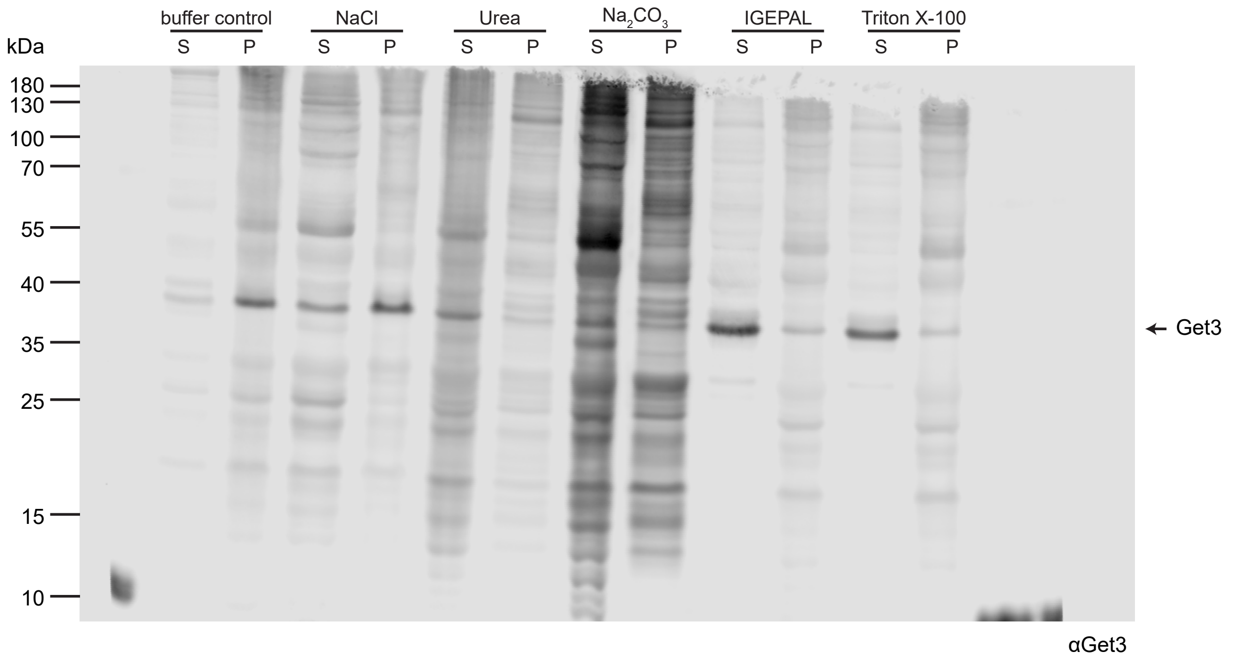

Figure 3E
Replicate 3

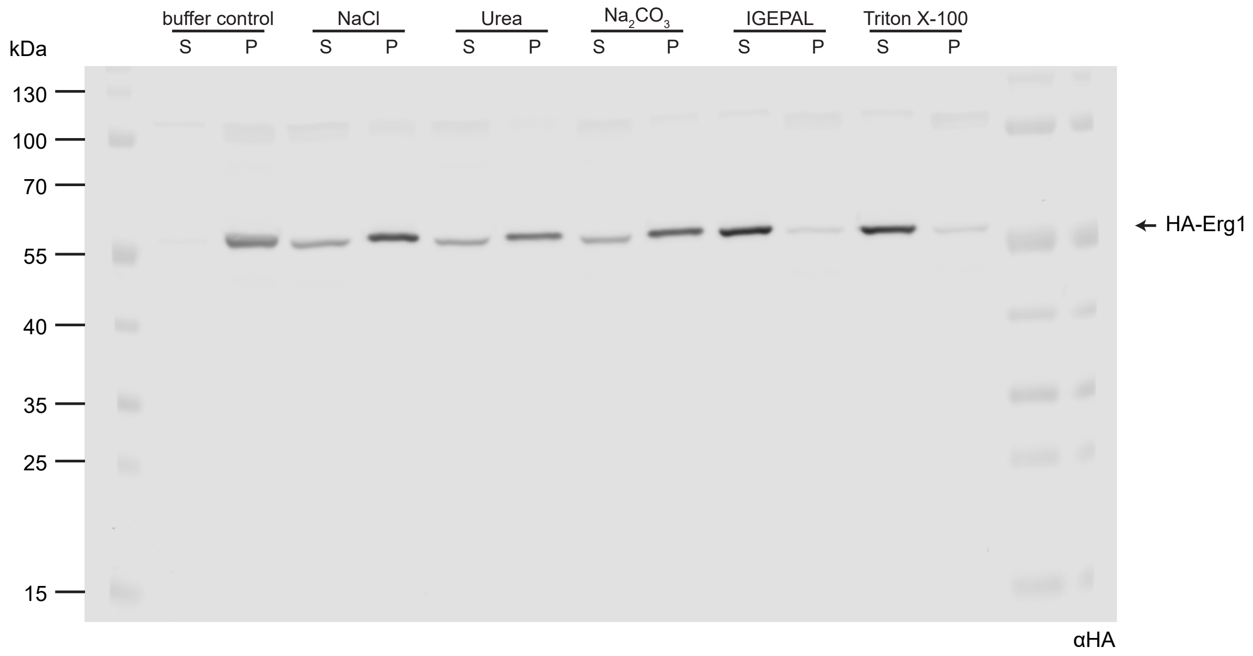

Figure 3E

Replicate 3

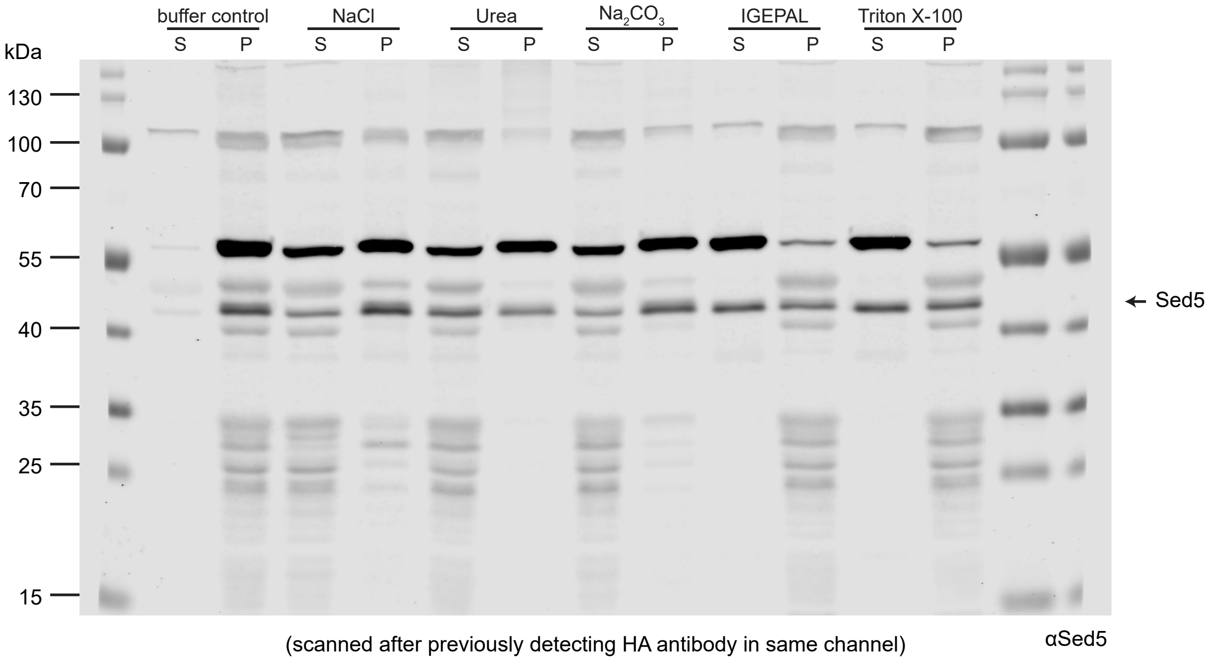

(scanned after previously detecting HA antibody in same channel)

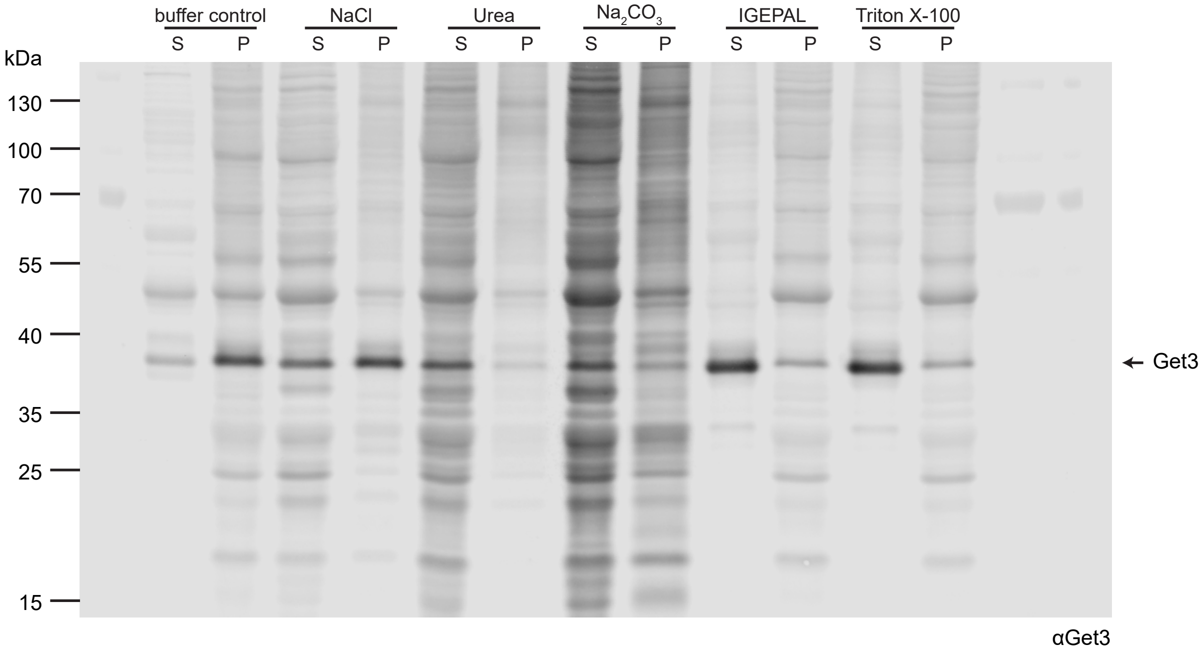

Figure 3F

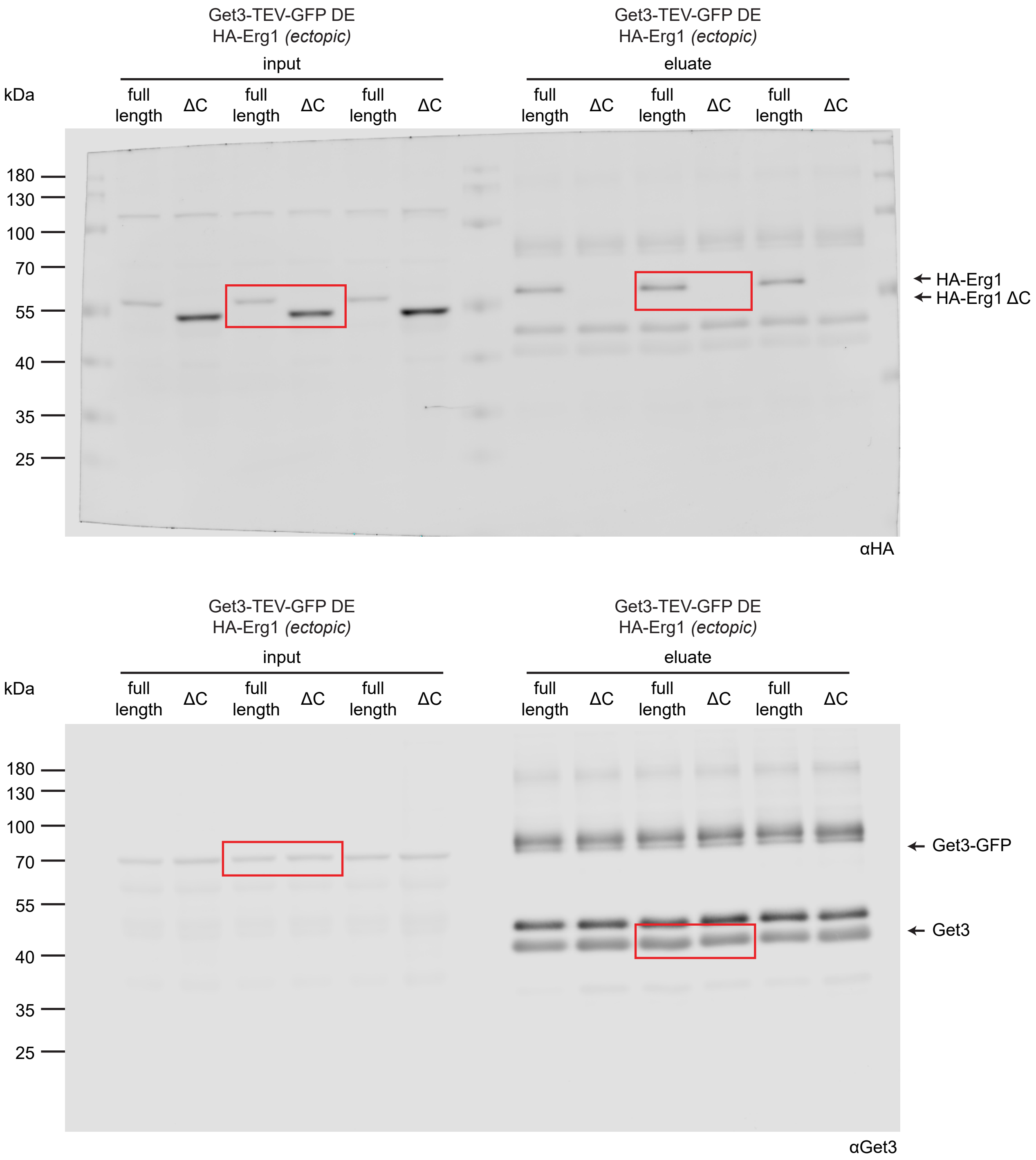
Get3-TEV-GFP DE
HA-Erg1 *(ectopic)*
input
Get3-TEV-GFP DE
HA-Erg1 *(ectopic)*
eluate
kDa
full length
ΔC
full length
ΔC
full length
ΔC
full length
ΔC
full length
ΔC
full length
ΔC
180
130
100
70
55
40
35
25
← HA-Erg1
← HA-Erg1 ΔC
αHA
Get3-TEV-GFP DE
HA-Erg1 *(ectopic)*
input
Get3-TEV-GFP DE
HA-Erg1 *(ectopic)*
eluate
kDa
full length
ΔC
full length
ΔC
full length
ΔC
full length
ΔC
full length
ΔC
full length
ΔC
180
130
100
70
55
40
35
25
← Get3-GFP
← Get3
αGet3

Figure 3H

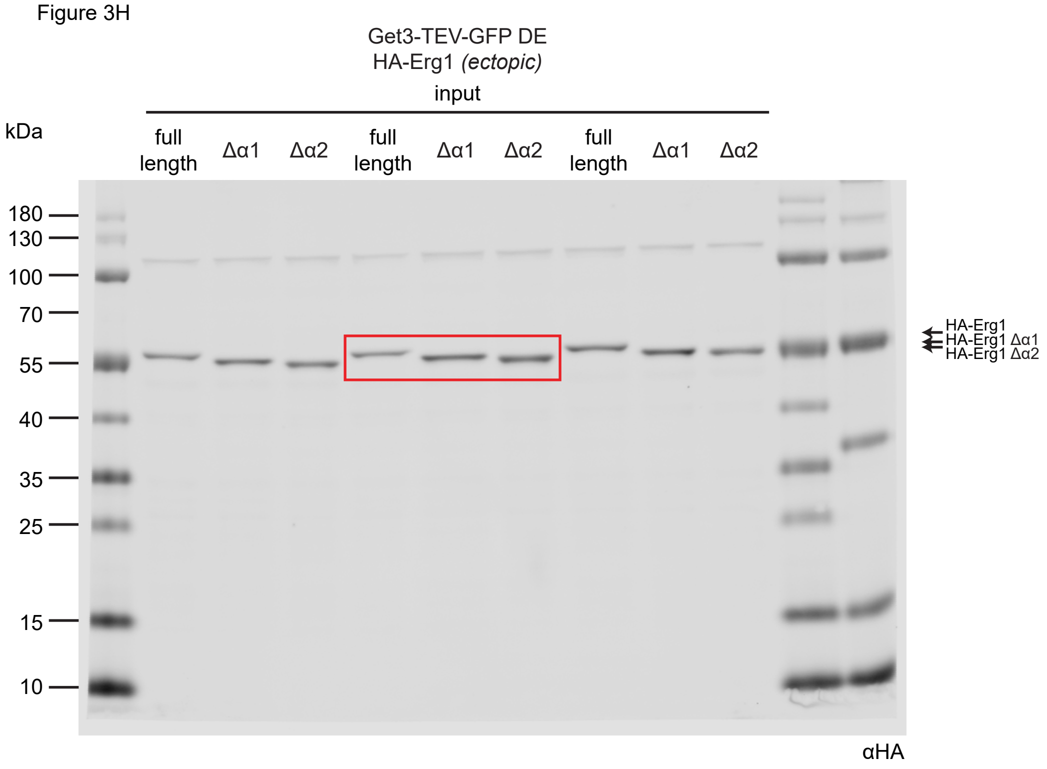
Get3-TEV-GFP DE
HA-Erg1 *(ectopic)*
input
kDa
full length
Δα1
Δα2
full length
Δα1
Δα2
full length
Δα1
Δα2
180
130
100
70
55
40
35
25
15
10
HA-Erg1
HA-Erg1 Δα1
HA-Erg1 Δα2
αHA

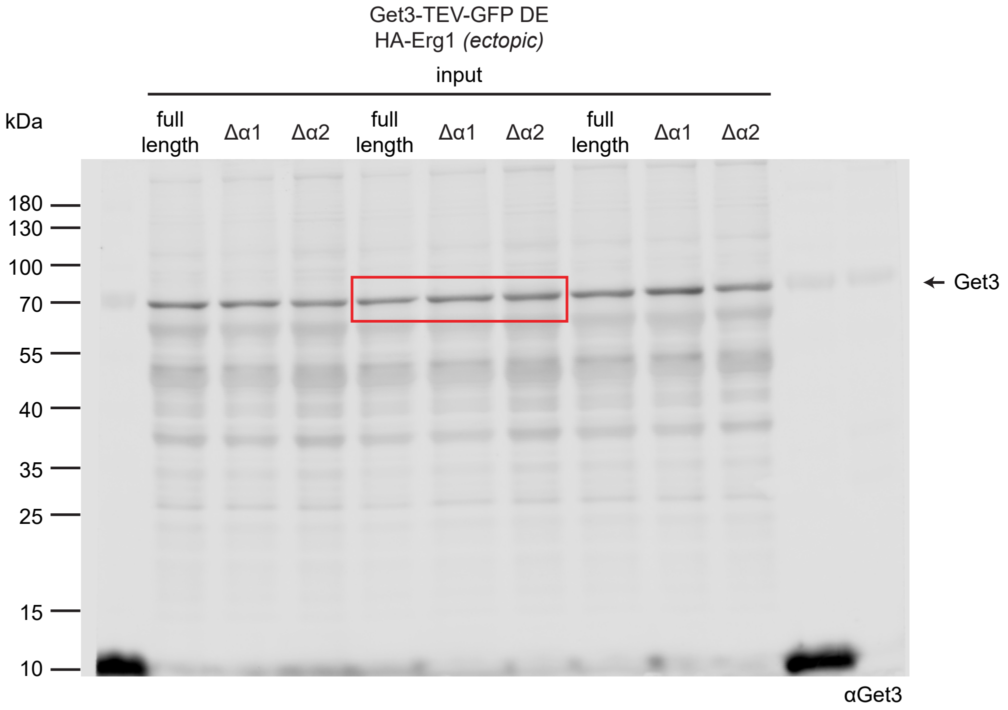
Get3-TEV-GFP DE
HA-Erg1 *(ectopic)*
input
kDa
full length
Δα1
Δα2
full length
Δα1
Δα2
full length
Δα1
Δα2
180
130
100
70
55
40
35
25
15
10
Get3
αGet3

Figure 3H

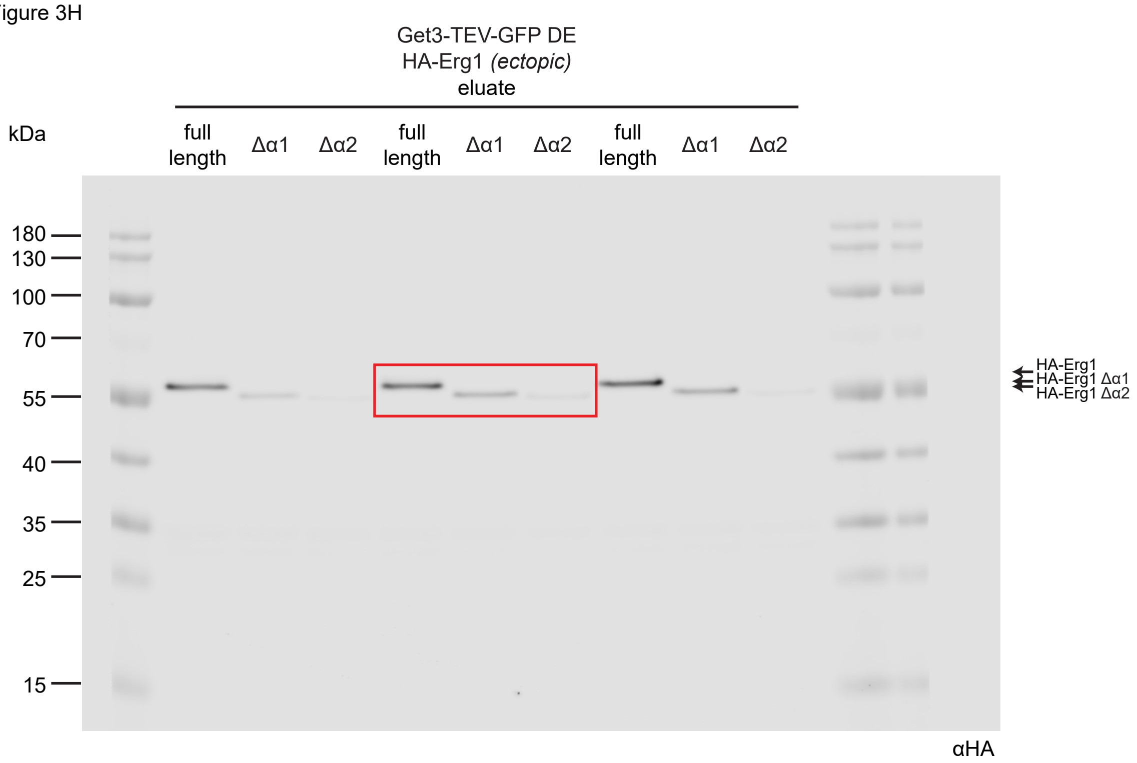
Get3-TEV-GFP DE
HA-Erg1 (ectopic)
eluate
kDa
full length
Δα1
Δα2
full length
Δα1
Δα2
full length
Δα1
Δα2
180
130
100
70
55
40
35
25
15
HA-Erg1
HA-Erg1 Δα1
HA-Erg1 Δα2
αHA

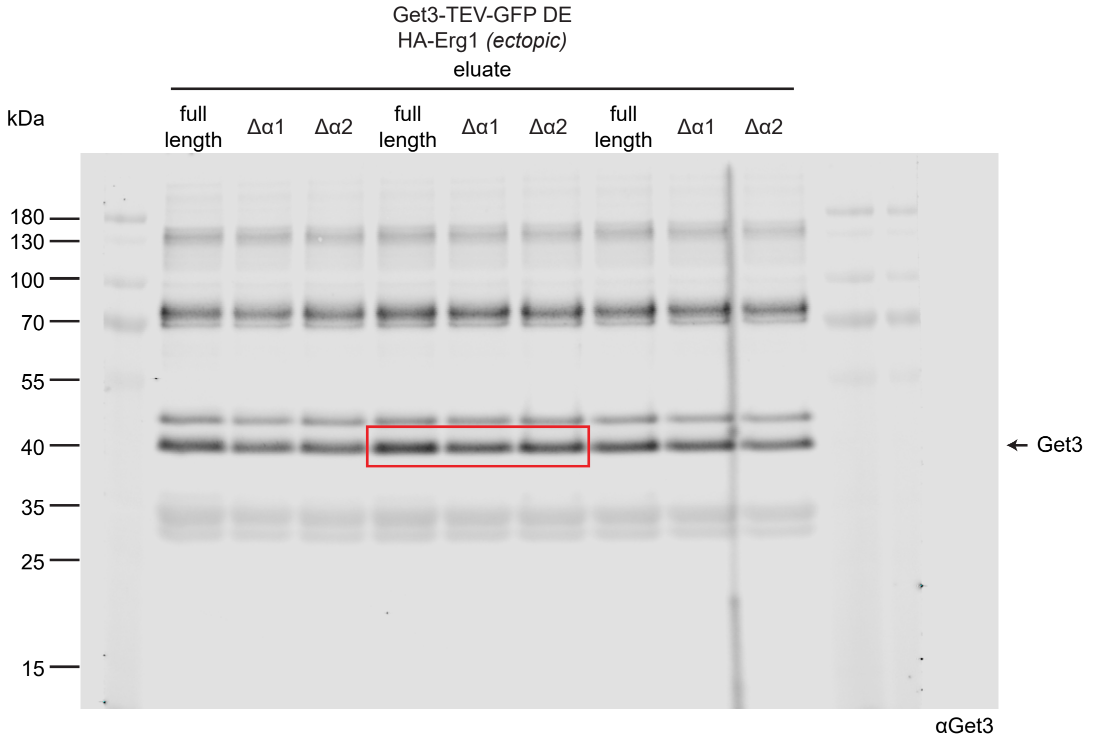
Get3-TEV-GFP DE
HA-Erg1 (ectopic)
eluate
kDa
full length
Δα1
Δα2
full length
Δα1
Δα2
full length
Δα1
Δα2
180
130
100
70
55
40
35
25
15
Get3
αGet3
